# 

**Published:** 1784

**Authors:** 


### PROMOTIONS.

Lately, Charles Blagden, M. D. S. R. S. to be a correſponding member of the Royal Academy of Sciences at Paris.—Dr. Alexander Stevenſon, of Glaſgow, and Dr. Andrew Duncan, of Edinburgh, to be phyſicians to his Royal Highneſs the Prince of Wales for Scotland.—Dr. Henry Cullen to be one of the phyſicians to the Infirmary at Edinburgh.—Dr. Frank to be profeſſor of phyſic at Gottingen.—Dr. Weickard, phyſician to the Prince Biſhop of Fulde, to be phyſician to the Empreſs of Ruſſia.

1784. *Jan.* 17. Dr. F. J. Voltelen to be profeſſor of chemiſtry at Leyden.

*Sept.* Dr. Robert Willan to be phyſician to the Finſbury Diſpenſary in London, in the room of Dr. Hopſon, who reſigned.—14. Dr. Thomas Lauth to be extra profeſſor of phyſic at Straſburgh—20. Meſſieurs John M'Morran, Hugh M'Dermot, William O'Dwyer, Henry M'Lagan, and William Major Dixon, to be doctors doctors of physic in the University of Glasgow.*—29. Dr. William Watson, and Dr. Richard Saunders, to be Fellows of the Royal College of Physicians, London.

*Oct.* 1. Mr. Richard Fletcher to be surgeon of the 5th regiment of dragoons, in the room of Mr. Robert Smithwick.—2. Mr. William Wood, late surgeon of the 96th, to be surgeon of the 78th regiment of foot.—9. Mr. John Weir to be surgeon to the troops at Jamaica.—Mr. West Hill to be surgeon to the troops at St. Vincent's.—11. Dr. Nicholas Paradys to be professor of physic at Leyden.

*Nov.* 24. Dr. F. Milman to be physician extraordinary, and Daniel Gib, Esq; to be surgeon extraordinary to the King's Houshold.

*Dec.* 11. Dr. Richard Dennison to be one of the physicians of the London Lying-in Charity.—15. Mr. Henry Cline to be surgeon to St. Thomas's Hospital, in the room of the late Mr. Thomas Smith.—23. Gilbert Blane, M.D. to be F. R. S.

* For the names of the gentlemen promoted to the degree of Doctor of Physic at Edinburgh, in the years 1783 and 1784, see the Quarterly Catalogue at the end of the present Volume.

### DEATHS.

Lately, at Paris, Mr. James Daran, one of the Surgeons in ordinary to the King of France. He was born at St. Frajon in Gaſcony, March 6, 1701, and after ſerving for ſome time as a Surgeon of the army, fixed his reſidence at Marſeilles, where he acquired ſo much reputation by the uſe of his bougies in obſtructions of the urethra, that the late M. de la Peyronie invited him to Paris. His practice in that metropolis was ſo conſiderable, that in the courſe of a few years he is ſaid to have amaſſed upwards of two millions of livres; but ſome unſucceſsful projects, in which he afterwards engaged, deprived him of almoſt the whole of this vaſt fortune. His *Obſ. Chirurg. ſur les Maladies de l'Urethre*, which have gone through ſeveral editions, were firſt publiſhed in 1745. He was alſo the author of a Treatiſe on the Gonorrhœa, printed at Paris in 1756; and a few years before his death, he communicated to the public his method of preparing bougies.—At Tunbridge Wells, in Suſſex, Mr. Henry Kipling, Surgeon.

1782. At Breflaw, in Silefia, Michael Morgenbeffer, M. D. fenior member of the College of Phyficians of that city.

1783. *Aug.* 10. At Caffel, J. Philip Berchelmann, M. D.

*Nov.* 18. At Leipfic, aged 63, Ant. Ridiger, M. D.

1784. *May.* In the ifland of Tobago, aged 35, Mr. William Wallace, member of the Corporation of Surgeons of London.

*July.* At Briftol Hot-wells, of a pulmonary confumption, aged 40, Mr. Thomas Meremoth, of Sandwich, in Kent, Surgeon.—At Chatenoi, in Franche Comté, aged 49, Francis Xavier Girod, M. D. Phyfician at Befançon, and member of the Royal Medical Society at Paris.

*Sept.* 1. At Nifmes, aged 81, John Francis Seguier, a celebrated Botanift and Antiquary. His father, who was an advocate at Nifmes, defigned him for his own profeffion; but when he was a young man, Signor Scipio Maffei, an Italian Nobleman, prevailed on him to accompany him to Verona, where he paffed twenty-five years, and during his refidence there publifhed his *Bibliotheca Botanica* * and his *Plantæ Veronenfes* †, two works in great eftimation

* 4to. 1740. † 8vo. 3 vols. 1745-54.

among among botaniſts. Several years before his death, he made a preſent of his library and muſeum to the Academy of Sciences at Niſmes, of which he had for many years been ſecretary; and in return for this benefaction, as well as from the regard which they thought due to his extraordinary merit, the Academy, after the deceaſe of the late Biſhop of Niſmes, elected him to be their Preſident.—12. At Straſburgh, of an apoplexy, George Lauth, M. D.—19. At Paiſley, near Glaſgow, Mr. William Stuart, Surgeon.—29. At Lambeth, Mr. Robert Carſon, Surgeon.

*Oct.* At Paris, Francis Bernard, Doctor Regent of the Faculty of Phyſic; and J. J. Peter de la Haye, M. D.—At Sudbury, in Suffolk, Mr. John Deeks, Surgeon.—20. At Leiceſter, aged 48, James Macvie, M. B. in the Univerſity of Louvaine, and one of the Phyſicians of the Infirmary at Leiceſter.

*Nov.* At Wentworth, in Yorkſhire, Stephen Simpſon, M. D.—At Preſton, in Lancaſhire, Mr. William Barton, Apothecary.—27. At Stamford, in Lincolnſhire, Mr. Price, Apothecary.—29. At Camberwell, in Surrey, aged 59, Mr. Thomas Smith, ſenior Surgeon of St.

Thomas's

Thomas's Hoſpital, and one of the Court of Aſſiſtants of the Corporation of Surgeons of London.

*Dec.* 3. At Aſhford, in Derbyſhire, William Bullock, M. D. in the commiſſion of the peace for the counties of Derby and Stafford, and author of an Inaugural Diſſertation *de Podagra*, printed at Leyden in 1765.—14. Tho. Younge, M. D. Phyſician at Sheffield.

---

### SECTION IV.

### QUARTERLY CATALOGUE.

1. AN Eſſay to inveſtigate the cauſes of the general mortality by Fevers, deduced from the knowledge of the nature of the blood, and the circulation: with miſcellaneous obſervations on ancient and modern writers. By *W. Charſley*, M. D. 8vo. *Kearſley*, London, 1783. 87 pages. 1s. 6d.

2. A ſhort Eſſay on the nature and ſymptoms of the Gout, with a view to recommend a medicine to the attention of thoſe who are afflicted with with that species of it, called the irregular or flying gout. By *James Rymer*, Surgeon. 8vo. *Evans*, London, 1784. 4d.

3. A Letter from a medical gentleman in town to his friend in the country; containing an authentic account of the difference between the Medical Society of Crane-Court, and Dr. Whitehead, during the late canvass for a Physician to the London Hospital. With a true copy of all the papers, both written and printed, which have passed between the contending parties. 8vo. *March*, London, 1784. 31 pages. 6d.

4. An Essay on the prevention of an evil, highly injurious to health, and inimical to enjoyment. By *William Edmonstone*, late Surgeon to the 89th regiment. 8vo. *Faulder*, London, 1784. 2s.

5. A short Treatise on the plant called Goose Grass, or Clivers, and its efficacy in the cure of the most inveterate scurvy. With the recipe for preparing and taking this simple and excellent medicine; and references to those cured of the disorder. By *John Edwards*, F. S. A. 8vo. *Dixwell*, London, 1784. 1s.

6. Dissertatio medica inauguralis de Amenorrhœa. Auctore *Rob. Harding*, Hiberno. 8vo. Edin. 1783. p. 43.

7. Disser-

7. Dissertatio Inauguralis de Scorbuto. Auctore *Ric. Kierman*, Hiberno. 8vo. Edin. 1783. p. 35.

8. Dissertatio Inauguralis de Mensibus, paucisque morborum ab eorum vitiis pendentium. Auctore *J. F. Martly*, Hiberno. 8vo. Edin. 1783. p. 43.

9. Dissertatio Medica Inauguralis de Tetano. Auctore *Gulielmo Munro*, Scoto. 8vo. Edin. 1783. p. 66.

10. Dissertatio Inauguralis de Ictero. Auctore *Joanne Murphy*, Hiberno. 8vo. Edin. 1783. p. 38.

11. Dissert. Inaug. de Ascite. Auctore *Ja. Nasmyth*, Scoto. 8vo. Edin. 1783. p. 20.

12. Dissert. Inaug. de Contagione. Auctore *Hug. Owen*, Cambro-Britanno. 8vo. Edin. 1783. p. 42.

13. Dissert. Inaug. de iis quæ ad Sanitatem conservandam plurimum conferre videntur. Auctore *Chrift. Stanger*, Anglo. 8vo. Edin. 1783. p. 37.

14. Diff. Medica Inaug. de Malo Hypochondriaco. Auctore *Joanne Stark*, A. M. Fifano. 8vo. Edin. 1783. p. 55.

15. Diff. Inaug. de Typho. Auctore *Thom. Waller*, Britanno. 8vo. Edin. 1783. p. 36.

16. Diff. Inaug. de Electricitate. Auctore *Abr. Wilkinſon*, Britanno. 8vo. Edin. 1783. p. 61.

17. Diff. Inaug. de Diæta ſeu Materia Diætetica. Auctore *Th. Bell*, Hiberno. 8vo. Edin. 1783. p. 48.

18. Diff. Inaug. de febribus intermittentibus medendo. Auctore *F. Buchanan*, Scoto. 8vo. Edin. 1783. p. 80.

19. Diff. Inaug. de Somno. Auctore *Roberto Cleghorn*, Scoto. 8vo. Edin. 1783. p. 51.

20. Diff. Inaug. de Scarlatina Cynanchica. Auctore *A. Coomby*, Scoto. 8vo. Edin. 1783. p. 55.

21. Diff. Inaug. de Somno. Auctore *Stephano Dickſon*, Hiberno. 8vo. Edin. 1783. p. 37.

22. Diff. Inaug. de Viſu. Auctore *J. Dudley*, Hiberno. 8vo. Edin. 1783. p. 33.

23. Diff. Inaug. de Variolis. Auctore *P. G. Fergus*, Hiberno. 8vo. Edin. 1783. p. 29.

24. Differt. Inaug. de Hyſteria. Auctore *Rob. Groat*, Britanno. 8vo. Edin. 1783. p. 49.

25. Diff. Inaug. de Hydro-Thorace. Auctore *Carolo Hill*, Hiberno. 8vo. Edin. 1783. p. 35.

26. Diff.

26. Diss. Inaug. de Ictero. Auctore *D. Keogh*, Hiberno. 8vo. Edin. 1783. p. 35.

27. Diss. Inaug. de Utero Gravido. Auctore *Benj. Kissam*, Americano. 8vo. Edin. 1783. p. 35.

28. Diss. Inaug. de Evaporatione. Auctore *J. Paterson*, Britanno. 8vo. Edin. 1783. p. 52.

29. Diss. Inaug. de Hydrope. Auct. *A. G. Robertson*, Antiguense. 8vo. Edin. 1783. p. 33.

30. Diss. Inaug. de Sonis et Auditu. Auctore *Edm. Somers*, Hiberno. 8vo. Edin. 1783. p. 35.

31. Diss. Med. Inaug. de Inflammatione. Auctore *Joanne Barclay*, Scoto. 8vo. Edin. 1784. p. 39.

32. Diss. Med. Inaug. de Morbo Psoadico. Auctore *R. Beckwith*, Anglo. 8vo. Edin. 1784. p. 38.

33. Diss. Med. Inaug. de Cancro. Auctore *Jonath. Clerke*, Hiberno. 8vo. Edin. 1784. p. 32.

34. Dissertatio Physico-Medica Inaug. de Voce Humana. Auctore *Edv. Long Fox*, Anglo-Britanno. 8vo. Edin. 1784. p. 47.

35. Diss. Med. Inaug. de Opio. Auctore *Edw. Harrison*, Anglo. 8vo. Edin. 1784. p. 47.

36. Diss. Med. Inaug. de Phthisi Pulmonali Idiopathica. Auctore *Ric. Kentish*, Anglo. 8vo. Edin. 1784. p. 45.

37. Diss. Med. Inaug. de Corde, et Sanguinis Circuitu. Auctore *Joanne Lawson*, Anglo. 8vo. Edin. 1784. p. 21.

38. Diss. Inaug. de Morbo Venereo. Auctore *Joanne Murdoch Logan*, Americano. 8vo. Edin. 1784. p. 50.

39. Differt. Physico-Medica Inaug. de Pathematibus animi eorumque in Corpus humanum effectibus. Auctore *Græmio Mercer*, Scoto. 8vo. Edin. 1784. p. 45.

40. Diss. Med. Inaug. de Sedentariæ Vitæ malis. Auctore *Joanne Potter*, Anglo. 8vo. Edin. 1784. p. 56.

41. Differt. Inaug. de Raphania. Auctore *Mich. Ryan*, Hiberno. 8vo. Edin. 1784. p. 41.

42. Differt. Inaug. de Asthmate Spasmodico. Auctore *Thom. Ryan*, Hiberno. 8vo. Edin. 1784. p. 39.

43. Differt. Medica Inaug. de Leucophlegmatia.

matia. Auctore *Joanne Unthank*, Hiberno. 8vo. Edin. 1784. p. 82.

44. Differt. Med. Inaug. de Vermibus Inteftinorum. Auctore *Thoma Wallis*, Hiberno. 8vo. Edin. 1784. p. 53.

45. Differtatio Therapeutica Inauguralis de Sudore. Auctore *Henrico Brounker Wilfon*, ex Infula Sancti Chriftophori. 8vo. Edin. 1784. p. 38.

46. Differt. Inaug. de Phrenitide Vera. Auctore *And. Berry*, Scoto. 8vo. Edin. 1784. p. 35.

47. Differt. Medica Inaug. de Febribus Intermittentibus. Auctore *Jacobo Collier*, Britanno. 8vo. Edin. 1784. p. 39.

48. Differt. Inaug. de Humorum Affimilatione. Auctore *Jacobo Curry*, Hiberno. 8vo. Edin. 1784. p. 33.

49. Differt. Inaug. de Hæmorrhagia Pulmonum. Auctore *Jacobo Donovan*, Hiberno. 8vo. Edin. 1784. p. 22.

50. Differtatio Inaug. de Aere fixo, five Acido Aereo. Auctore *Thoma Addis Emmet*, Hiberno. 8vo. Edin. 1784. p. 60.

51. Differtatio Pathologica Inauguralis de Sanguinis per corpus vivum circulantis putredine. Auctore *Samuele Ferris*, Anglo-Britanno. 8vo. Edin. 1784. p. 122.

52. Dif-

52. Dissertatio de Febre Puerperarum. Auctore *Thoma Fulhame*, M. A. Hiberno. 8vo. Edin. 1784. p. 12.

53. Differt. Inaug. de Differentiis inter Fœtum et Adultum. Auctore *Jacobo Gerard*, Anglo. 8vo. Edin. 1784. p. 49.

54. Differt. Inaug. de Vita Marina. Auctore *Gulielm. Henderson*, Britanno. 8vo. Edin. 1784. p. 57.

55. Dissertatio Physico-Chemica, Inauguralis, de Principio Sorbili, sive communi mutationum Chemicarum Causa, Quæstionem, an Phlogiston sit substantia, an qualitas, agitans; et alteram Ignis Theoriam complectens. Auctore *Ricardo Lubbock*, Anglo-Britanno. 8vo. Edin. 1784. p. 137.

36. Differt. Inaug. de Submersis. Auctore *Jacobo M'Donnell*, Hiberno. 8vo. Edin. 1784. p. 52.

57. Differtatio Inaug. de Pulsu Arteriarum. Auctore *Alex. Pellisier*, Hiberno. 8vo. Edin. 1784. p. 29.

58. Differt. Inaug. de Amenorrhœa. Auctore *Thoma Spens*, Edinburgense. 8vo. Edin. 1784. p. 33.

59. Differt. Inaug. de Cute et Tactu. Auctore *Laurent. Walsh*, Hiberno. 8vo. Edin. 1784. p. 40.

60. Differ-

60. Dissertatio Inauguralis Quædam de Typho Complectens. Auctore *Joanne M'Morran*, Chir. Hiberno. 8vo. Glasguæ, 1784. p. 57.

61. *Floris Jacobi Voltelen* Oratio aditialis de Chemiæ hodiernæ pretio rite constituendo, Lugduni Batavorum, in Academica Panegyri, a. d. 17 Januarii, 1784, recitata, quum Extraordinariam Medicinæ et Chemiæ publice docendæ Provinciam Solenniter Capesseret. 4to. Ludg. Batav. 1784. p. 53.

62. *Nicolai Paradys* Oratio de Diligenti Therapeutices Universalis Studio, maximo recte medendi instrumento. Publice habita a. d. 11 Octobris, 1784, quum ordinariam Medicinæ Professionem in Academia Lugduno-Batava Auspicaretur. 4to. Lugd. Batav. 1784. p. 24.

63. Experimentum Anatomicum, quo Arteriolarum Lymphaticarum existentia probabiliter adstruitur, institutum, descriptum, et icone illustratum, a *Jano Bleuland*, M. D. 4to. Lugd. Batav. 1784. p. 36.

64. Tentamen Medicum Inaugurale de usu Corticis Peruviani in Morbis Hydropicis. Auctore *Antonio Henrico van Noehuys*, Sylvæ ducensi. 4to. Lugdun. Batav. 1782. p. 42.

65. Spe-

65. Specimen Medicum Inaugurale, continens Hepatitidis Hiſtoriam. Auctore *Martino Urbano Van Yperen*. Moſa-Slufenſi. 4to. Lugdun. Batav. 1782. p. 35.

---

IN addition to the Caſe of fractured Scull, deſcribed in a former part of this volume (page 278), we think it right to obſerve, that the mode of treatment adopted in that caſe was firſt ſuggeſted by Mr. Wilmer, an ingenious Surgeon at Coventry, in his *Caſes and Remarks in Surgery*, publiſhed in 1779. In that work he contends, that it is not abſolutely neceſſary to remove any portion of the ſcalp, even when the ſcull is extenſively fractured; and in proof of this, he obſerves, that in ſeveral caſes of fractured ſcull, in which it was neceſſary to apply the trephine more than once, he has ſeen the cure accompliſhed, without removing any portion of the ſcalp. Mr. Wilmer adds, that the wounds were healed in half the time that muſt have been employed if exciſion had taken place; and that he has ſeen but one caſe, thus treated, where the exfoliation of the bone was neceſſary.—The caſe related by our correſpondent merits attention, as it affords an additional proof of the ſafety and efficacy of the method of cure recommended by Mr. Wilmer.

INDEX.

### I N D E X.

### A.

Page

AIKIN, J. de lactis secretione in puerperis, — 328

Analogy, botanic, attempt to recommend the study of, 218

Anatomy, system of, from Monro, Winslow, &c. — 104

Arvidson, P. A. de chemiæ progressu, — — 110

### B.

Banks, Sir Joseph, his *Reliquiæ Houstounianæ*, — 253

Barclay, J. de inflammatione, — — 435

Bartolozei, F. physiological experiments by, — 222

Baths, Russian, an account of — — 355

Baumer, J. W. fundamenta chemiæ, — 336

Beccaria, Padre, elogio del, — — 111

Beckwith, R. de morbo psoadico — — 435

Bell, Tho. de diæta, — — 434

Bergman, Sir Thorb. outlines of mineralogy, — 106

——— de analysi lithomargæ, — 110

——— terra absestina, — ibid.

——— chemiæ progressu, — ibid.

——— his physical essays, English translation of, 219

Berkenhout, Dr. J. on the hydrophobia — 326

Berry, And. de phrenitide vera, — — 437

Bew, George, his experiments on latent heat, — 205

Blagden, Dr. Charles, on the congelation of quicksilver, 233

Bleuland, Janus, de arteriolarum lymphaticarum existentia, 439

Bonafide, F. l'inoculazione de Vajuolo, — — 221

Bonnet, Charles, œuvres d'histoire naturelle, — 222

Brambilla, J. A. instrumentarium chirurgicum, — 331

Buchanan, F. de febribus intermittentibus, — 434

Butter, Dr. W. on arteriotomy, — — 327

### C.

Cammel, Mr. case of extra-uterine fœtus — 396

Camper, P. over de steenen in de pisblaas, — 111

Cavendish, Henry, on the congelation of quicksilver, — 230

Cevadilla, natural history of, — — 135

Chambon, de Montaux, M. des maladies des femmes — 224

Chandler, Dr. B. case of a stone of the bladder, — 387

Charsley, Dr. W. on the mortality by fevers, — 431

Chavasse, N. on the cure of chronic dysentery, — 297

Chaulnes, Duc de, on the fusible salt of urine, — 225

Cleghorn, R. de Somno, — — 434

Clerke, J. de cancro, — — — 435

Colds and coughs, address to the public concerning, — 103

Collier, J. de febribus intermittentibus — 437

Comus, Sieur, his experiments in medical electricity, — 58

Coomby, A. de scarlatina cynanchica, — 434

Cornette, M. chemical essays by, — 346, 347, 352

Cribb, W. case of luxation of the thigh, — 412

Cullen, Dr. E. his translation of Bergman's essays, — 219

Page

Curry, J. de humorum aſſimilatione, — 437

Curtis, W. catalogue of plants, — — 220

D.

Daniel, Dr. S. caſe of painful menſtruation, — 183

Deaſe, W. his account of a fatal caſe of ganglion, — 172

Deaths, — — 100, 211, 318, 428

Denman, Dr. on the ſpontaneous evolution of the fœtus, 64, 301

Dewell, T. philoſophy of phyſic, — — 104

Dickſon, S. de Somno, — — 434

Dijon, Nouveaux mem. de l'acad. de, — 133

Dillon, P. on the cure of fiſtula in ano by cauſtic, — 392

Donly, H de veſicantium uſu, — — 328

Donnell, J. Mac, de ſubmerſis, — — 438

Donovan, J. de hæmorhagia pulmonum, — — 437

Dropſy, ſovereign remedy for, — — 106

Dudley, J. de viſu, — — 434

Duncan, Dr. his abridgement of Hoffman's practice of phyſic, 160

Durande, M. Flore de Bourgogne, — 109

——— on the ſolvent of gallſtones, — 126, 134

Durer, Seb. de acidulis freudenthalenſibus, — 336

Dyſentery, obſervations on, — — 137, 375

E.

Edmonſtone, W. on the prevention of gonorrhœa, — 432

Edwards, John, on the efficacy of gooſe graſs, — ibid.

Elſner, C. F. Beytrage zur fieberlehre, — — 112

Emmet, Tho. Addis, de aere fixo, — — 437

Epilepſy, obſervations on, — — 58, 361

F.

Fergus, P. G. de variolis, — — 434

Ferris, S. de ſanguinis putredine, — — 437

Firenze, conſtituzione epidemica di, — 224

Fontana, abbé, on poiſons, — — 1, 416

Fox, E. L. de voce humana, — — — 435

Fulhame, T de febre puerperarum, — 438

Fuller, J. hints for reſtoring animation, — — 328

G.

Gall ſtones, ſolvent of, — — 126, 134

Ganglion, fatal caſe of, — — 172

Gardane, M. on the colic of ſeamen, — 228

Garlick, W. his account of two diſſections, — 186

Gaubius, J. D. anecdotes of, — — 119

Gerard, J. de differentiis inter fœtum et adultum, — 438

Gillibert, J. on the virtues of certain plants, — 129

Gout, remarkable cure of, — — 86

Gregory, Jac. conſpectus medicinæ theoreticæ, — 382

Groat, Rob. de hyſteria, — — 434

Guaraldi, Hieron. opuſcula anatomica, — 224

H. Page

Hamilton, Dr. R. on the efficacy of opium in gangrene, — 75, 190

Harding, Rob. de amenorrhoea, — — 432

Harrison, Edw. de opio, — — 436

Harwood, Rev. Dr. case of, — — 328

Hemlock dropwort, instance of its poisonous effects, — — 192

Henderson, Gul. de vita marina, — — 438

Henry, Thomas, his experiments on latent heat, — 89

——— translation of M. Lavoisier's essays, — 159

Hodson, Thomas, his obs. on Stern's medical advice, — 103

Hoffman, F. abridgement of his medicina rationalis systematica, 161

Hortus Uptoniensis, — — 108

Houlston, Dr. Thomas, his account of a mineral spring at Wigan, 311

——— obs. on poisons, and on the use of mercury in dysentery, — — 374

——— letter to, from a physician in Italy, — 419

Houlston, William, case of injury of the brain, — 292

Houstouni reliquiæ, — — 253

Howard, John, on the cure of hydrocele by Seton, — 61

Hulke, William, case of remarkable spasmodic affection, 389

Hydrophobia, cases of, and obs. relative to, 85, 220, 314, 354, 421

I.

Jacob, Edward, case of luxation of the os femoris, — 312

Influenza of 1782, account of, — — 335

Jones, William, case of a fractured scull, — 278

Itch, new remedy for the cure of, — — 350

Jussieu, Joseph de, anecdotes of, — — 337

K.

Kentish, R. de phthisi pulmonali idiopathica, — 436

Keogh, D. de ictero, — — 435

Kierman, R. de scorbuto, — — 433

Kippis, Rev. Dr. his life of Sir John Pringle, — — 140

Kirkland, Dr. Tho. on the present state of medical surgery, 384

Kirwan, R. on the attractive power of the mineral acids, 29

Kissam, B. de utero gravido, — — 435

L.

Landriani, M. on phlogisticated alkali and Prussian blue, — 222

Languth, J. F. de catarrho epidemico A. 1782, — 335

Lauro-cerasus, remarks on, — — 18

Lawson, J. de Corde, et Sanguinis circuitu, — 436

Leveling, H. M. de atmosphæræ pressione varia, — 336

Lewis, Dr. W. his abridgement of Hoffman's practice of physic, 160

Lieberkuhn, J. N. dissertationes anatomicæ, — — 217

Lieutaud, Joseph, anecdotes of, — — 117

Linné, Charles von, supplementum plantarum, — 256

——— anecdotes of, — — 329

Logan, J. M. de morbo venereo, — — 436

Lubbock, Ric. de principio sorbili — — 438

Ludwig, C. F. de suffusionis per acum curatione — 329

M. Page

Macqueen, Dr. Malcolm, on angina pectoris, —— 162

Magellan, J. H. de, description of his glass apparatus, &c. 108

Magnetism, animal, works relative to, 266, 332, 333, 334

—— medical, inquiries relative to, —— 364

Majon, Bened. pharmacopeia manualis, — — 336

Mann, Abbé, history of his recovery from the gout, —— 86

Maret, M. on the solvent of gall stones, —— 134

Martineau, Mr. case of dropsy of the ovarium, —— 315

Martinet, M. obs. sur les cancer, —— —— 109

Martly, J. F. de Mensibus, —— — 433

Matthews, Stephen, on hepatic diseases, —— 104

Matthiis, M. de, on the cure of the hydrophobia, —— 220

Mederer, M. J. J. de rabie canina, — — 224

Mercer, G. de pathematibus animi, —— 436

Mercury, its efficacy in dysenteries depending on a diseased liver, 375

—— large quantity of, swallowed with impunity, — 387

—— congelation of, papers relative to, 227, 230, 233

Michaelis, Dr. F. on hydrophobia, *page* 286. On the decussation of the optic nerves —— —— 289

Moore, James, his method of diminishing pain in surgical operations, 369

Morozzo, Count, his letter to M. Macquer, —— 221

Morran, J. Mac, de Typho, — — 439

Moscati, J. B. metodo per curare l'idropisia, —— 336

—— P. sul sangue fluido e rappresso, —— — 223

Moss, William, on the management of children, —— 324

—— medical survey of Liverpool, —— 327

Munro, G. de Tetano, —— — 433

Murphy, J. de ictero, —— —— ibid.

Mursinna, C. L. on the dysentery, —— 137

Mynors, Robert, remarks on a case of fractured scull, — 284

N.

Nasmyth, J. de ascite, —— —— 433

Nerves, reproduction of, experiments relative to, — 23

—— structure of, as examined with a microscope, —— 25

—— optic, decussation of in quadrupeds, —— 289

Newman, J. W. on the principles of the medical profession, 105

Noohuys, A. H. Van, de usu cort. Peruv. in morb. hydropicis, 439

O.

Odier, M. on the hydrocephalus internus, —— 354

Owen, Hugo, de contagione, —— — 433

P.

Pain in surgical operations, mode of preventing, —— 369

Palfyn, Jean, eloge de, — —— 329

Paris, memoirs of the royal medical society at, 113, 248, 348

—— academy of sciences at, —— 337

Paterson, J. de evaporatione, —— — 435

Pearson, Dr. G. on the waters of Buxton, —— 220

Pellisier, Alex. de pulsu arteriarum, —— —— 438

Page

Peterſburgh, memoirs of the Imperial academy at, — 129

Pharmacopeia Edinenſis, — — 44

Phlogiſton, experiments relative to, — — 238

Pitcairne, Dr. Arch. life of, — — 326

Platina, ſhewn to be not a perfect metal, — — 344

Plenk, J. J. pharmacologia chirurgica, — 109

Poiſons, obſervations on, — 1, 193, 374, 416

Potter, J. de ſedentariæ vitæ malis, — — 436

Prieſtley, Dr. J. his experiments relative to phlogiſton, — 238

Pringle, Sir John, anecdotes of, — — 140

Promotions, — — 98, 209, 316, 426

Pulteney, Dr. R. on the poiſonous effects of hemlock dropwort, 192

Q.

Queſtions, prize, 79, 80, 81, 82, 199, 200, 201, 309, 310, 413, 414

R.

Reill, J. C. de polycholia, — — 223

Roberts, Benj. caſes of three women inoculated during pregnancy, 399

Robertſon, Dr. R. on the jail fever, — — 219

——— A. G. de hydrope, — — 435

Robſahm, C. G. de terra abſeſtina, — — 110

Roſa, Chevalier, letters concerning his doctrine, — 223

Ryan, Mich. de raphania, — — 436

—— Thom. de aſthmate ſpaſmodico, — ibid.

Rymer, James, on the ſcurvy, page 103. On the gout, 432

S.

Samoilowitz, M. ſur la peſte, — — 330, 331

Sanchez, Antonio Nunes Ribeiro, life of, — 92

——— on the Ruſſian baths, — 355

Sarcocele, ſpecies of in Senegal, deſcribed, — 32

Schotte, Dr. on the ſarcocele of Senegal, — — ibid.

——— ſynochus atrabilioſa, — 260

Sheldon, John, hiſtory of the abſorbent ſyſtem, — 157

Sherwin, Mr. on the marine ſcurvy, — — 325

Soaps, acid, uſe of and mode of preparing, — 352

Somers, Edm. de ſonis et auditu, — — 435

Spens, Thom. de amenorrhœa, — — 438

Spleen, caſe of enlargement of — — 186

Stackens, J. C. Hebammenunterricht in Geſprachen, — 112

Stanger, C. de ſanitate, — — 433

Stark, J. de malo hypochondriaco, — — ibid.

Sympathy defended, — — 214

Symphyſis pubis, ſection of performed, — 313, 415

Syſtema vegetabilium of Linné, Engliſh tranſlation of, — 107

T.

Fana, Count, his eulogy of father Beccaria, — 111

Thouret, M. on the cranium of the fœtus, — — 363

Tillet, M. on platina, — — 344

Toxicodendron, experiments relative to the poiſonous effects of, 20

Tranſactions, philoſophical, — — 29, 226

Vater,

V.

Page

Vater, C. de præſagiis vitæ et mortis, —— 224

Viper, poiſon of, remarks on, —— — 3, 416

Vicq D'Azyr, M. obſervations by, —— 127, 340

Vogel, A. F. chirurg. Wahrnehmungen, —— 336

Voice, organ of, its ſtructure, —— — 340

Voltelen, F. J. de chemiæ hodiernæ pretio, —— 439

U.

Unthank, J. de Leucophlegmatia, —— 437

Urine, fuſible ſalt of, manner of preparing it, —— 225

Uterus, gravid, lectures on, —— —— 328

———— laceration of, inſtances of, —— 424

W.

Walker, George, on the minute ſhells found near Sandwich, 218

—— Dr. Joſhua, on the waters of Harrogate and Thorp arch, ibid.

Wall, Dr. Martin, on ſelect ſubjects of chemiſtry and phyſic, 105

Waller, Tho. de typho, —— — 433

Wallis, Tho. de vermibus inteſtinorum, —— 437

Walſh, Laurent. de cute et tactu, —— —— 438

Water, decompoſition of, —— —— 202

Webſter, Dr. Charles, his life of Dr. Pitcairne, —— 326

White, Charles, on a ſwelling of the lower limbs in lying in women, 50

—— Dr. Robert, on cancers, —— —— 70

Whitehead, Dr. letter concerning his diſpute with a medical ſociety, 432

Whitehurſt, John, caſe of palſy cured by electricity, — 77

Wilkinſon, Abr. de electricitate, —— —— 434

Willan, Dr. Robert, caſe of obſtruction of the bowels, —— 401

Wilmer, Mr. his manner of treating fracture of the ſcull, — 440

Wilſon, Andrew, on diſeaſes of children, —— 105

Wilſon, H. B. de Sudore, —— —— 437

Withering, Dr. W. his tranſlation of Bergman's mineralogy, 106

Y.

Yperen, M. U. Van, de hepatitide, —— — 440

Z.

Zarda, A. V. pharmaca vegetabilia, — — 109
Zorn, John, plates of medicinal plants, —— 221

